# Yearly changes in cases of acute acquired comitant esotropia during a 12-year period

**DOI:** 10.1007/s00417-023-06047-8

**Published:** 2023-04-17

**Authors:** Yoichi Okita, Akiko Kimura, Akiko Masuda, yoshihito Mochizuki, Miho Kondo, Katsuhide Yamadera, Fumi Gomi

**Affiliations:** Department of Ophthalmology, Hyogo Medical University, 1-1, Mukogawa-Choho, Nishinomiya-Cityity, Hyogo, Japan

**Keywords:** Acute acquired comitant esotropia, Yearly changes, Smartphone, Age, Refractive error, Bielschowsky type

## Abstract

**Purpose:**

The number of patients with acute acquired comitant esotropia (AACE) has been increasing in Japan. The purpose of this study was to investigate the changes in the number and characteristics of patients with AACE examined in our institution during a 12-year period.

**Methods:**

We retrospectively reviewed the medical records of patients with AACE aged < 30 years who suddenly developed diplopia or esotropia and were examined in Hyogo College of Medicine Hospital from January 2008 to December 2021. We investigated the association of the yearly changes in the number of patients with the age category, refractive error category, AACE type, esotropia type, and use or nonuse of smartphones.

**Results:**

The total number of patients with AACE was 171, and this number significantly increased each year (Pearson correlation coefficient, 0.9450; *p* < 0.0001). Significant increases were found among students in junior high school and beyond, patients with myopia, patients with Bielschowsky type AACE, and patients with basic esotropia (*p* < 0.0001 for all). We compared two age groups, elementary school students and below versus junior high school students and above, and found that the rate of increase was significantly higher in the junior high school students and above (estimate, 1.951; *p* < 0.0001), and the non-myopia group and myopia group and found that the rate of increase was significantly higher in the myopia group (estimate, 1.891; *p* < 0.0001). Excessive use of smartphones was confirmed in 82 of 133 patients, and the rate of the increase in the number of patients with AACE was significantly greater among patients with than without excessive use of smartphones (estimate, 1.098; *p* = 0.0009).

**Conclusion:**

This study confirmed a significant increase in the number of patients with AACE in recent years. The excessive use of smartphones may be associated with the increase in AACE.


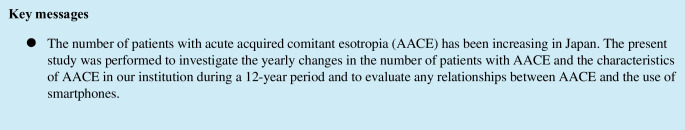


## Introduction

In Asian countries (including Japan), unlike in Western countries, exotropia is much more common than esotropia in patients with comitant strabismus [1]. However, the frequency of acute acquired comitant esotropia (AACE) has recently shown an increasing tendency, especially in younger generations. Although AACE has been considered a rare condition, recent reports indicate that excessive use of digital devices can trigger AACE [2, 3]. With the widespread use of smartphones and other digital devices, the number of patients with AACE is increasing [2, 4]. In 2016, Lee et al. [2] described 12 patients with AACE caused by excessive use of smartphones, and the esotropia was significantly improved in 9 of these patients by restriction of smartphone use. This suggests the possibility of a relationship between excessive use of smartphones and development of esotropia.

The present study was performed to investigate the yearly changes in the number of patients with AACE and the characteristics of AACE in our institution during a 12-year period and to evaluate any relationships between AACE and the use of smartphones.

## Patients and methods

This retrospective single-center study was performed in compliance with the Declaration of Helsinki and approved by the Research Ethics Review Committee of Hyogo Medical College (Approval No. 202104-756). We reviewed the medical records of patients with AACE examined at the Department of Ophthalmology in Hyogo College of Medicine Hospital from January 2008 to December 2021 and extracted the data of patients who developed onset of diplopia from 2008 to 2019.

The diagnostic criteria for AACE in this study were partially modified from previous reports [2, 5] as follows: (1) known date of onset of diplopia or esotropia, (2) no limitation of ocular motility, (3) no particular abnormalities on cranial and orbital computed tomography or magnetic resonance imaging, (4) absence of accommodative component, (5) no history of systemic disease or head trauma, and (6) no history of ocular problems such as strabismus or amblyopia. We included patients who met the following criteria: age of < 30 years, corrected visual acuity of 20/20 in both eyes, and follow-up of ≥ 1 month. We excluded patients who showed a reduction in esotropia when wearing hyperopic glasses, had an unknown date of symptom onset, had convergence spasms, and had decompensated esotropia.

The following background information of the patients was investigated: sex, age at onset, age at first visit to our hospital, period from onset of diplopia to first visit to our hospital, refractive error, angle of esotropia, and type of AACE. We asked the patients about their daily duration of smartphone use and identified excessive use as > 4 h of smartphone use per day.

The angle of esotropia was measured by the alternate prism cover test at near (33 cm) and distance (5 m), and stereopsis for near was measured by the Titmus stereo test (Stereo Optical Inc., Chicago, IL, USA). Spherical equivalent (SE) refractive error (spherical power + 1/2 cylindrical power) was measured after administration of cyclopentolate hydrochloride in patients aged < 12 years and after administration of tropicamide/phenylephrine in patients aged > 13 years. Automated assessment of refraction was performed by an autorefractometer (RC-5000; Tomey Inc., Nagoya, Japan).

We evaluated the yearly changes in the number of patients diagnosed with AACE and the background characteristics of the patients throughout the study period. The age categories were based on the classification of 6 years of elementary school (6–11 years old), 3 years of junior high school (12 to 14 years old), and 3 years of senior high school (15 to 17 years old) in the Japanese educational system. The patients’ age at onset was categorized into the following five categories: < 5 years, 6–11 years (elementary school students), 12–14 years (junior high school students), 15–17 years (senior high school students), and > 18 years. Because the smartphone penetration rate in Japan rapidly increases among junior high school students and above, we categorized the patients into two additional groups: elementary school students and below and junior high school students and above.

Refractive error was categorized into hyperopia [+ 2.00 to + 0.50 diopter (D)], emmetropia (+ 0.25 to − 0.25 D), low myopia (− 0.50 to − 2.75 D), mild myopia (− 3.00 to − 5.75 D), and severe myopia (greater than − 6.00 D).

AACE was classified into three main types as reported previously [ 6–8]: (1) Swan type, which occurs following unilateral occlusion or vision loss; (2) Burian–Franceschetti type, which is thought to be caused by physical and psychological stress in the absence of an accommodative component; and (3) Bielschowsky type, which develops in patients with myopia of − 5.00 D or higher and is characterized by divergence insufficiency esotropia. In this study, patients with refractive error of less than − 5.00 D but with under-correction or no correction of myopia were also classified as having Bielschowsky type AACE.

The difference between near and distance esodeviation was categorized into the following three types: (1) divergence insufficiency type [≥ 10 prism diopter (PD) larger at distance than at near], (2) basic type (difference of < 10 PD between near and distance), and (3) non-accommodative convergence excess type (≥ 10 PD larger at near than at distance).

## Statistical analysis

The two independent variable comparisons in the study population were performed with Student’s t-test for continuous variables approaching a normal distribution and with the Mann–Whitney U test for continuous non-normally distributed variables.

Pearson’s correlation test was used to identify any correlation between variables, and simple and multiple linear regression analyses were used to predict dependent variables.

Statistical analysis was performed using JMP Pro version 14.0.0 (SAS Institute Inc., Cary, NC, USA). For significance testing, a risk rate of < 5% was considered statistically significant.

## Results

### Patients’ background characteristics (Table 1)

**Table 1:** Patients’ background characteristics

characteristics	2008	2009	2010	2011	2012	2013	2014	2015	2016	2017	2018	2019
case:[women, men]	3[1,2]	4[0, 4]	4[0, 4]	10[5]	12[4, 8]	13[5, 8]	12[3, 9]	18[9]	18[8, 10]	21[10, 11]	32[11, 21]	24[10, 14]
age: [years]	13.7	13.5	12.3	17.2	15.2	15.8	15.6	17.5	17.2	17.2	18.4	17.5
age at onset: [years]	12.3	13.0	8.5	15.3	13.2	14.0	14.0	15.3	15.4	16.3	17.3	16.5
SE refractive error: [diopters]	-2.33	-0.94	-1.81	-3.60	-2.25	-2.34	-1.85	-3.15	-3.49	-4.07	-4.80	-4.15
period from the onset of diplopia to the first visit: [months]	17	10	47	24	23	22	18	27	22	13	14	12
angle of esotropia at near: [prism diopters]	28.3	30.5	44.3	37.2	36.6	36.7	38.3	39.7	30.6	31.0	30.3	29.8
angle of esotropia at distance: [prism diopters]	31.7	32.0	43.5	33.5	41.1	37.8	38.9	38.9	30.8	31.6	31.5	28.5

The total number of patients aged > 12 years was 171. Male patients (*n* = 104, 60.8%) were more common than female patients (*n* = 67, 39.2%). The mean ± standard deviation age at symptom onset was 15.4 ± 5.2 years (range, 2–29 years), and the median age was 15 years. According to the above-defined age categories, 5 (2.9%) patients were < 5 years old, 21 (12.2%) were elementary school students, 49 (28.7%) were junior high school students, 47 (27.5%) were senior high school students, and 49 (28.7%) were > 18 years old.

The mean age at the first visit to our hospital was 16.9 ± 5.7 years (range, 2–30 years). The mean period from the onset of diplopia to the first visit to our hospital was 18.6 ± 19.7 months (range, 0–105 months). The mean follow-up period at the hospital was 19.0 ± 20.9 months (range, 1–103 months).

At the first examination, the mean SE refractive error was − 3.46 ± 3.10 D (range, + 2.00 to − 13.25 D). By refractive error, the patients exhibited hyperopia (*n* = 13, 7.6%), emmetropia (*n* = 32, 18.7%), low myopia (*n* = 24, 14.0%), mild myopia (*n* = 61, 35.7%), and severe myopia (*n* = 41, 24.0%). Myopia was present in 126 (73.7%) of the patients. Anisometropia was present in 10 (5.8%) patients. The mean angle of esotropia was 33.5 ± 19.6 PD (range, 1–123 PD) at near and 33.9 ± 15.8 PD (range, 6–87 PD) at distance.

The classification of AACE was Swan type in 1 (0.8%) patient, Burian–Franceschetti type in 4 (2.3%), and Bielschowsky type in 166 (96.9%). Forty-eight (28.9%) of the 166 patients with Bielschowsky type had uncorrected or poorly corrected myopia. One hundred fifteen (69.3%) of the 166 patients had SE refractive error of less than − 5.00 D but received appropriate refractive correction and were not categorized into any classification of AACE. All 10 cases of anisometropia were of the Bielschowsky type. With respect to the esotropia classification, divergence insufficiency type was present in 27 (15.8%) patients, basic type in 128 (74.8%), and nonaccommodative convergence excess type in 16 (9.3%).

The amount of time spent using a smartphone per day was confirmed in 112 (65.5%) of 171 patients. Forty-three (38.4%) patients were female, and the mean age at onset was 16.0 ± 5.1 years (range, 2–28 years). Excessive use of smartphones was confirmed in 82 (73.2%) of the 112 patients. Among the remaining 30 patients, 9 patients used their smartphones for 3 h per day, 6 used their smartphones for. 2 hours, 4 used their smartphones for 1 h, and 11 did not use their smartphones.

### Time trends in characteristics

**Fig. 1:**
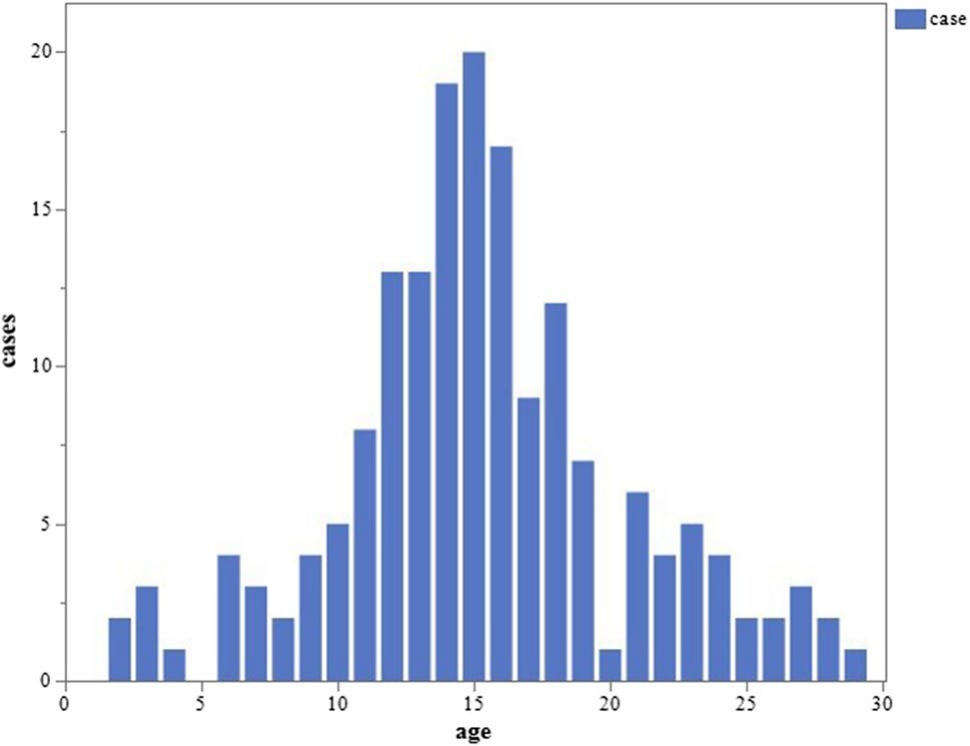
Yearly changes in number of patients with AACE. The number patients with AACE when counting the year of the patient’s onset significantly increased beginning in 2008: 3, 4, 4, 10, 12, 13, 12, 18, 18, 21, 32, and 24 patients (Spearman’s rho, 0.9450; *p* < 0.0001)

The number of patients with AACE significantly increased each year throughout the study period (from. 2008 to 2020) (r^2^= 0.8929, *p* < 0.0001, patients =  − 4625.248 + 2.304*year) (Fig. 1).

**Fig. 2:**
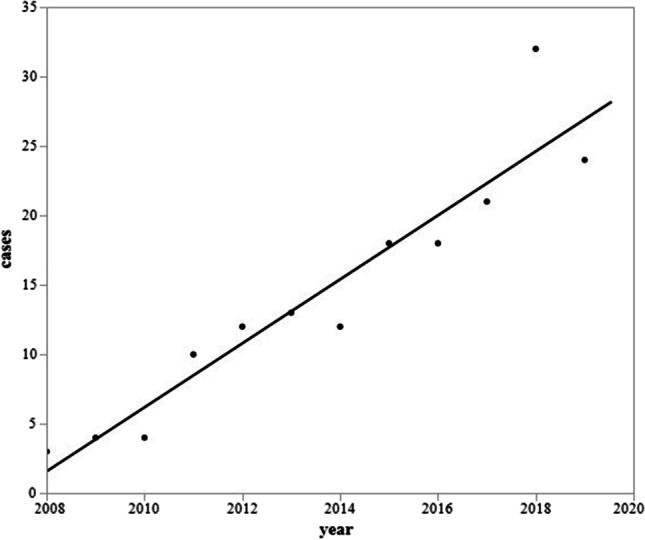
Yearly changes in number of patients with AACE by age classification. The rate of increase was significantly higher in junior high school students and above than in elementary school students and below (estimate, 1.951; *p* < 0.0001)

A similar analysis in each year group subcategory showed significant increases over time in all groups except for patients aged < 5 years [< 5 years (r^2^=0.1732, *p* = 0.1784, patients =  − 119.270 + 0.059*year), elementary school students (r^2^=0.4300, *p* = 0.0206, patients =  − 469.944 + 0.234*year), junior high school students (r^2^=0.3666, *p* = 0.0369, patients =  − 890.023 + 0.444*year), senior high school students (r^2^=0.5354, *p* = 0.0068, patients =  − 988.753 + 0.493*year), and > 18 years (r^2^=0.7198, *p* = 0.0005, patients =  − 2157.261 + 1.073*year)]. We compared two age groups, elementary school students and below versus junior high school students and above, and found that the rate of increase was significantly higher in the junior high school students and above (estimate, 1.951; *p* < 0.0001) **(**Fig. 2**)**.

**Fig. 3:**
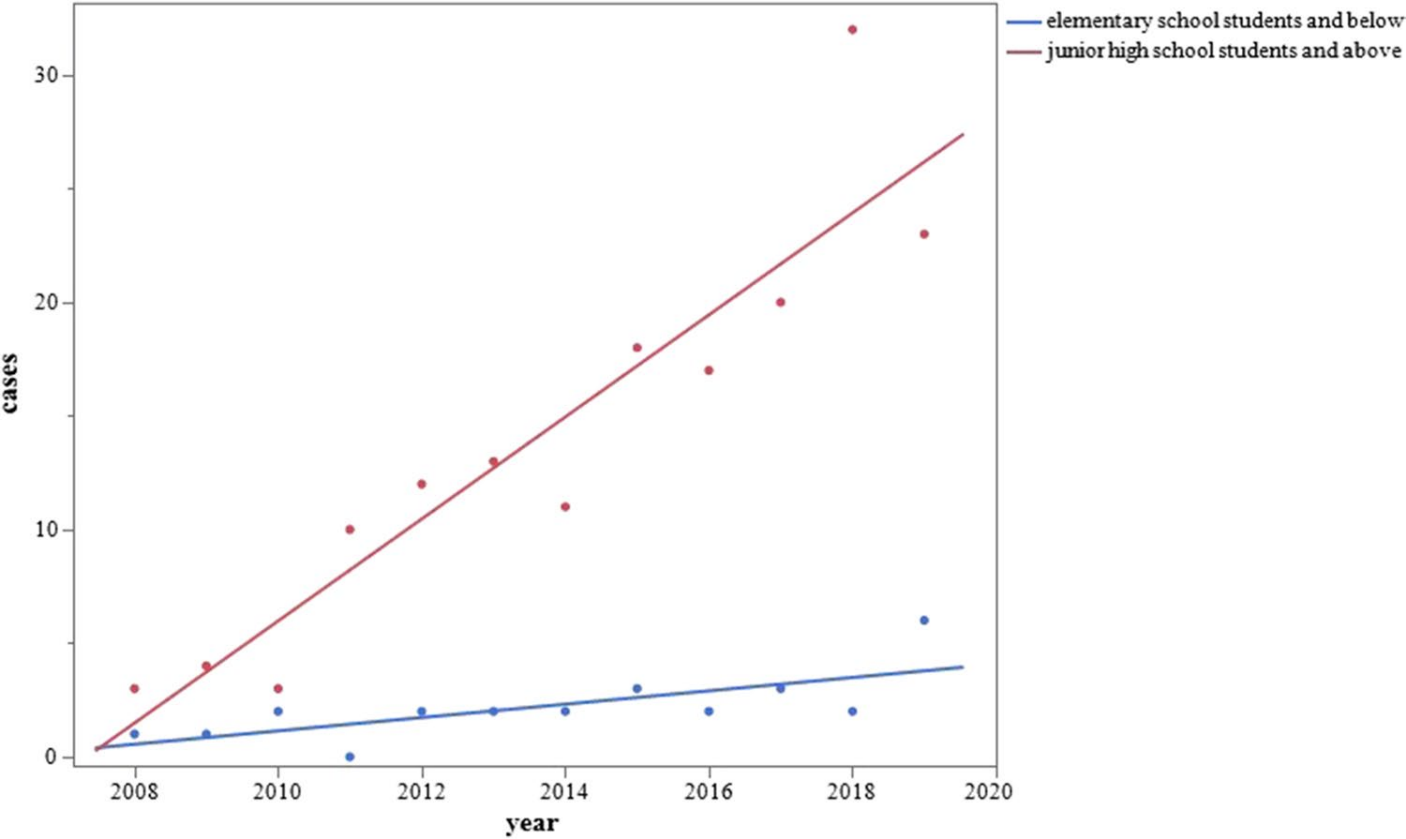
Yearly changes in number of patients with AACE by refractive error classification. The rate of increase was significantly higher in the myopia group than in the non-myopia group (estimate, 1.951; *p* < 0.0001)

In each refractive error subcategory, all groups of patients except those with emmetropia showed significant increases [hyperopia group (r^2^=0.5718, *p* = 0.0044, patients =  − 456.530 + 0.227*year), emmetropia (r^2^=0.0017, *p* = 0.8983, patients = 44.908 +  − 0.021*year), low myopia (r^2^=0.5473, *p* = 0.0060, patients =  − 842.825 + 0.420*year), mild myopia (r^2^=0.8121, *p* < 0.0001, patients =  − 1593.044 + 0.794*year), and severe myopia (r^2^=0.6052, *p* < 0.0029, patients =  − 1777.756 + 0.885*year)]. We also compared the non-myopia group and myopia group and found that the rate of increase was significantly higher in the myopia group (estimate, 1.891; *p* < 0.0001) (Fig. 3).

**Fig. 4:**
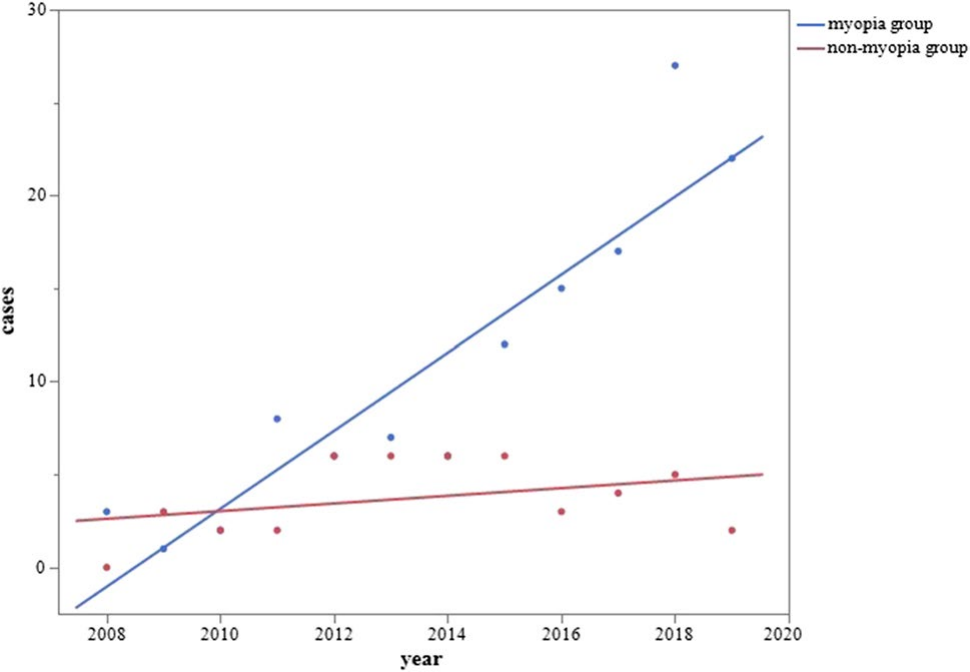
Yearly changes in number of patients with AACE by Bielschowsky type. The rate of increase was not significantly different between − 5.00 D or more and less than − 5.00 D among patients with Bielschowsky type (*p* = 0.8895)

Among the AACE types, only the Bielschowsky type showed a significant increase [Swan type (r^2^=0.1545, *p* = 0.2063, patients =  − 63.279 + 0.031*year), Burian–Franceschetti type (r^2^=0.0, *p* = 1.0000, patients = 0.333 + 0*year), and Bielschowsky type (r^2^=0.8838, *p* < 0.0001, patients =  − 4632.705 + 2.3077*year)]. Within the Bielschowsky type, we compared patients with − 5.00 D or more and less than − 5.00 D and found no significant difference in the rate of increase between the two groups (*p* = 0.8895) [− 5.00 D or more (r^2^=0.7206, *p* = 0.0005, patients =  − 2283.818 + 1.136*year) and less than − 5.00 D (r^2^=0.9215, *p* < 0.0001, patients =  − 2348.887 + 1.171*year)] (Fig. 4).

Among the types of esotropia, the basic type and nonaccommodative convergence excess type showed significant increases [basic type (r^2^=0.9256, *p* < 0.0001, patients =  − 3762.886 + 1.874*year), divergence insufficiency type (r^2^=0.4815, *p* = 0.1130, patients =  − 413.122 + 0.206*year), and nonaccommodative convergence excess type (r^2^=0.3465, *p* = 0.0441, patients =  − 449.240 + 0.224*year)].

**Fig. 5:**
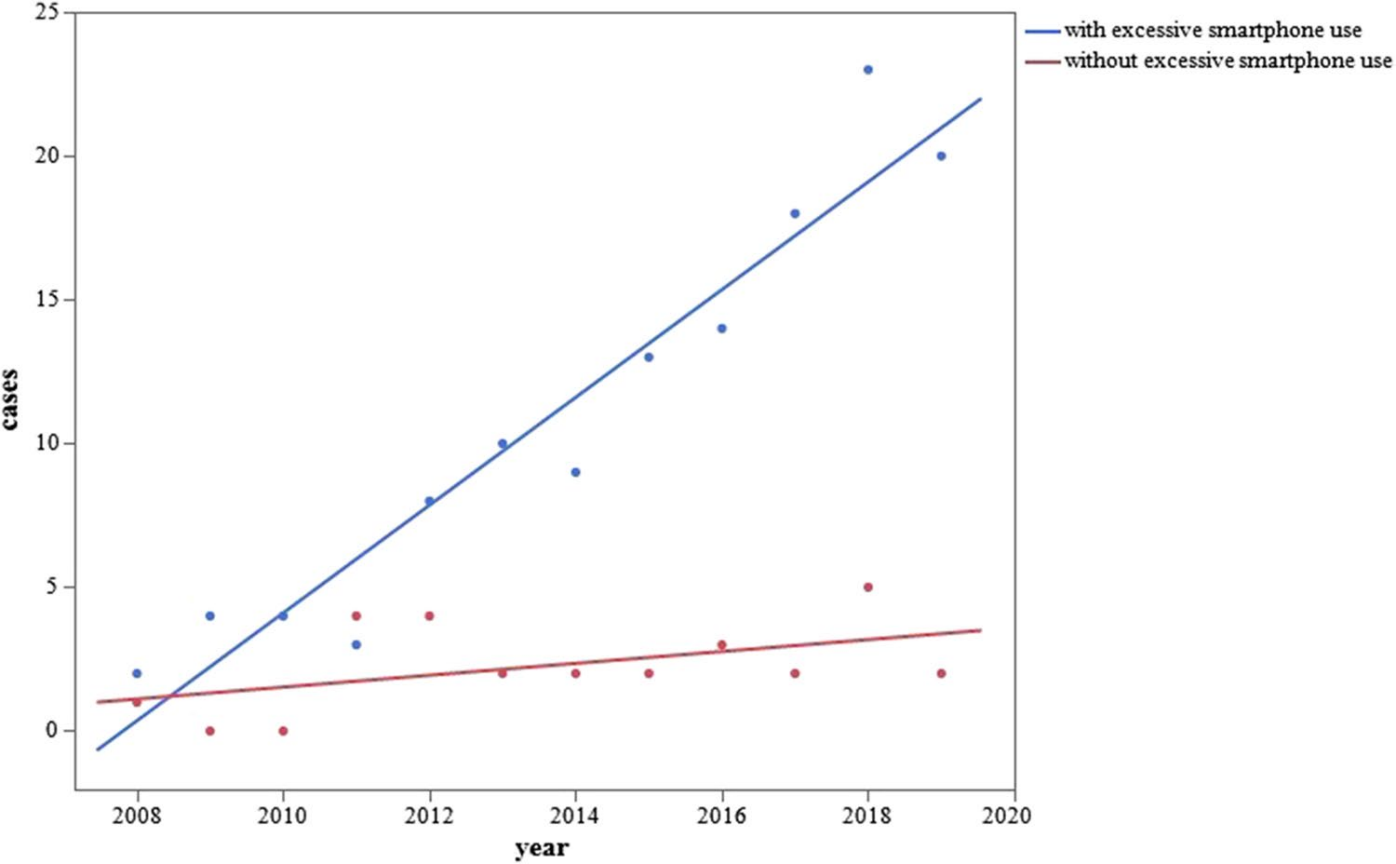
Yearly changes in number of patients with AACE according to excessive smartphone use. Multiple regression analysis showed a significantly higher annual increase in patients with than without excessive smartphone use (estimate, 1.098; *p* = 0.0009)

Finally, with respect to excessive smartphone use, patients with excessive smartphone use group showed a significant increase in the number of patients with AACE per year [with excessive smartphone use (r^2^=0.9256, *p* < 0.0001, patients =  − 3762.886 + 1.874*year) and without excessive smartphone use (r^2^=0.2318, *p* = 0.1130, patients =  − 413.122 + 0.206*year)]. Multiple regression analysis showed a significantly higher annual increase among the patients with excessive smartphone use (estimate, 1.098; *p* = 0.0009) (Fig. 5).

## Discussion

AACE is a rare condition [9, 10] and has been estimated to account for only 0.3% of childhood strabismus [7, 10] in the absence of hyperopia or exogeneous causes such as a brain tumor [11, 12] or total occlusion therapy [9, 11]. The classification of three types of mechanisms underlying the onset of AACE proposed by Burian and Miller [6] has commonly been used. More recently, however, research has indicated that prolonged use of digital devices such as smartphones at closely near may be one cause of AACE, especially in adolescents [2, 4]. In 2016, Lee et al. [2]. reported that the onset of AACE in 12 patients with an average age of 13.3 years (range, 7–16 years) was considered to be related to excessive use of smartphones because refraining from smartphone use decreased the degree of esodeviation in 9 of these patients.

In the present study, we retrospectively analyzed patients with AACE aged < 30 years who suddenly presented with diplopia or esotropia from 2008 to 2019 to determine whether the number of patients with AACE (especially younger patients) is increasing with the widespread use of digital devices such as smartphones. Because Ali et al. [13] reported that the mean age of eight patients with decompensated esotropia that caused by AACE was 29 years (range, 20–48 years), we analyzed patients aged ≤ 30 years to exclude those with decompensated esotropia. The purpose of this study was twofold: first, to determine the significance of the increase in patients with AACE, and second, to determine the relationship between excessive use of smartphones and the increase in patients with AACE.

We confirmed a significant increase in the number of patients with AACE over the 12-year study period. This study confirms that the number of patients with AACE, a growing research topic in recent years, has markedly increased. The peak age at onset in our study was 15 years. Iimori et al. [3] investigated patients with AACE aged 5 to 35 years from 2015 to 2018 and found a peak age at onset of 16 years, followed by 15 years, similar to the present study. The rate of increase in the number of patients with AACE was significantly different between elementary students and below and junior high school students and above, with the greatest increase in the number of patients with AACE in junior high school students (12–14 years), and the junior and senior high school students (12–17 years) accounting for about 60% of the cases.

In Japan, sales of smartphones started in 2008, and the penetration rate was about 10% in 2010; however, it steadily increased to 49.5% in 2012, 72.0% in 2015, and 83.4% in 2019. Digital devices such as smartphones have rapidly become widespread, especially among young people (Trends in Household Ownership of Information and Communication, Equipment, Ministry of Internal Affairs and Communications: https://www.soumu.go.jp/johotsusintokei/whitepaper/ja/r02/html/nd252120.html). The number of patients with AACE is increasing with the widespread use of digital devices such as smartphones [2, 4]. The penetration rate of smartphones among 13- to 19-year-olds in Japan increased from just under 60.0% in 2013 to 79.5% in 2017; in comparison, the rate among 6- to 12-year-olds was only about 20.0% in 2013 and 30.3% in 2017 (https://www.soumu.go.jp/johotsusintokei/whitepaper/ja/h30/html/nd142110.html).

A relationship between AACE and myopia has been reported [14, 15]. In the present study, the rate of increase in the number of patients with AACE was significantly higher among patients with than without myopia. In terms of the mechanism of AACE onset, Bielschowsky [14] indicated the involvement of uncorrected myopia because of the inability to maintain a balance between the converging and diverging forces of the eyes due to excessively close proximity to the targets. However, the 10 cases of adult-onset AACE described by Spierer [5] in 2003 did not fit into the previous types of AACE, and the common characteristics of these 10 patients were as follows: esotropia had developed after the age of 16 years, the angle of esodeviation was similar for distance and near, myopia was present in all but one patient, and normal stereopsis was regained after surgical correction of the esotropia. Our study also showed that 166 (96.9%) of the 171 patients had Bielschowsky type AACE, 69.3% of which were − 5.00 D or less, and we found no significant difference in the rate of increase in the number of patients with AACE between those with − 5.00 D or more and less than − 5.00 D.

Indeed, in this study, 61 (74.4%) of the 82 patients with AACE whose smartphone use time was confirmed had excessive smartphone use (≥ 4 h per day). In addition, in more recent years, the rate of increase in the number of patients with excessive smartphone use was significantly higher than that of patients without excessive smartphone use (*p* = 0.0009). Furthermore, remission of entropion was observed in four patients by refraining from using smartphones, suggesting a relationship between excessive smartphone use and the development of AACE, as reported by Lee et al. [2].

Lee et al. [2]. speculated that the excessive use of smartphones at near may overstimulate accommodation and vergence, causing the medial rectus (MR) muscle to become dominant; this in turn may result in low fusional divergence ability and latent esophoria, resulting in the development of manifest esotropia. Our study included an 18-year-old patient who developed AACE after watching three- dimensional attractions. We consider that out-of-the-ordinary dichoptic viewing, such as three- dimensional attractions, triggers vergence-accommodation conflict, and a large parallax stimulus becomes a convergence stimulus that exceeds relative convergence, resulting in not only disruption of fusion but also disruption of vergence-accommodation regulation. Therefore, the imbalance among vergence, accommodation, and convergence due to excessive use of smartphones at near may be involved in the onset of AACE rather than refractive error, even if uncorrected myopia is involved [3, 13].

Cai et al. [15]. first reported that the distance of the MR insertion from the limbus was shorter in patients with AACE than in patients with exotropia. It is assumed that the shorter distance of MR insertion may lead to strengthening of convergence tonus, resulting in an imbalance between convergence and divergence and subsequent esotropia. However, Lai et al. [16]. and Niyaz et al. [17] indicated that the distance of the MR insertion from the limbus was not significantly different among patients with esotropia, patients with exotropia, and control patients. Therefore, whether the shorter distance of the MR from the limbus is characteristically recognized only in patients with AACE needs to be examined in a larger population. Nevertheless, it is considered to be a possible predisposing factor for AACE.

This study has some limitations. First, because of the single-center and retrospective nature of our study, the data were limited to what was available in the patients’ records and the small proportions. We excluded patients with decompensated esotropia to focus only on patients with acquired and acute-onset diplopia or esotropia without a congenital element. Second, the duration of smartphone use per day was not confirmed for all patients. In addition, we were unable to confirm whether the distance was extremely close or not during use of the smartphones. Looking at smartphones at extremely close distances may be a risk factor for onset of AACE.

Vagge et al. [18]. described four patients with AACE who presented during the national lockdown due to COVID-19 in Italy. All patients spent 8 to 10 h a day using computers, tablets, and smartphones to play, access school lessons, and navigate social networks. The prolonged school closures and home confinement during the COVID-19 lockdown period resulted in important lifestyle behavioral changes, such as a significant increase in screen time [19], highlighting the risk of an increased myopia burden due to these new lifestyle habits [20]. However, it also indicates that excessive application of near vision might have other detrimental consequences, including the onset of AACE. This study was not affected by COVID-19 because it reviewed patients with AACE from 2008 to 2019. The transition of patients with AACE after 2020 should be noted in future research.

In Japan, multicenter research on AACE began in 2018, and we expect that much information on the relationship between digital devices and the onset of AACE will soon become available.
